# Effectiveness of Web-Based Versus Face-To-Face Delivery of Education in Prescription of Falls-Prevention Exercise to Health Professionals: Randomized Trial

**DOI:** 10.2196/jmir.1680

**Published:** 2011-12-22

**Authors:** Stephen Maloney, Romi Haas, Jennifer L Keating, Elizabeth Molloy, Brian Jolly, Jane Sims, Prue Morgan, Terry Haines

**Affiliations:** ^1^Department of PhysiotherapyMonash UniversityFrankstonAustralia; ^2^Southern HealthMelbourneAustralia; ^3^Health Professions Education and Educational ResearchMonash UniversityMelbourneAustralia; ^4^Health Workforce Education and Assessment ResearchMonash UniversityMelbourneAustralia; ^5^Healthy Ageing Research UnitMonash UniversityMelbourneAustralia

**Keywords:** Education, professional development, course design, distance education, students

## Abstract

**Background:**

Exercise is an effective intervention for the prevention of falls; however, some forms of exercises have been shown to be more effective than others. There is a need to identify effective and efficient methods for training health professionals in exercise prescription for falls prevention.

**Objective:**

The objective of our study was to compare two approaches for training clinicians in prescribing exercise to prevent falls.

**Methods:**

This study was a head-to-head randomized trial design. Participants were physiotherapists, occupational therapists, nurses, and exercise physiologists working in Victoria, Australia. Participants randomly assigned to one group received face-to-face traditional education using a 1-day seminar format with additional video and written support material. The other participants received Web-based delivery of the equivalent educational material over a 4-week period with remote tutor facilitation. Outcomes were measured across levels 1 to 3 of Kirkpatrick’s hierarchy of educational outcomes, including attendance, adherence, satisfaction, knowledge, and self-reported change in practice.

**Results:**

Of the 166 participants initially recruited, there was gradual attrition from randomization to participation in the trial (n = 67 Web-based, n = 68 face-to-face), to completion of the educational content (n = 44 Web-based, n = 50 face-to-face), to completion of the posteducation examinations (n = 43 Web-based, n = 49 face-to-face). Participant satisfaction was not significantly different between the intervention groups: mean (SD) satisfaction with content and relevance of course material was 25.73 (5.14) in the Web-based and 26.11 (5.41) in the face-to-face group; linear regression *P* = .75; and mean (SD) satisfaction with course facilitation and support was 11.61 (2.00) in the Web-based and 12.08 (1.54) in the face-to-face group; linear regression *P* = .25. Knowledge test results were comparable between the Web-based and face-to-face groups: median (interquartile range [IQR]) for the Web-based group was 90.00 (70.89–90.67) and for the face-to-face group was 80.56 (70.67–90.00); rank sum *P* = .07. The median (IQR) scores for the exercise assignment were also comparable: Web-based, 78.6 (68.5–85.1), and face-to-face, 78.6 (70.8–86.9); rank sum *P* = .61. No significant difference was identified in Kirkpatrick’s hierarchy domain *change in practice*: mean (SD) Web-based, 21.75 (4.40), and face-to-face, 21.88 (3.24); linear regression *P* = .89.

**Conclusion:**

Web-based and face-to-face approaches to the delivery of education to clinicians on the subject of exercise prescription for falls prevention produced equivalent results in all of the outcome domains. Practical considerations should arguably drive choice of delivery method, which may favor Web-based provision for its ability to overcome access issues for health professionals in regional and remote settings.

**Trial Registration:**

Australian New Zealand Clinical Trials Registry number: ACTRN12610000135011; http://www.anzctr.org.au/ACTRN12610000135011.aspx (Archived by WebCite at http://www.webcitation.org/63MicDjPV)

## Introduction

Continuing professional development (CPD) is an essential component of the educational continuum of health care professionals. Participation in continuing education is a hallmark of professional behavior [1,2] and a requirement for continued registration to practice for many professions. It is an imperative for continued advancement in the quality of health care and is a key educational activity for universities, professional organizations, and other educational institutions [2]. Effectively delivered CPD can narrow the gap between new evidence of best practice and current practice, enhancing practitioners’ knowledge and behaviors and positively influencing service delivery and patient outcomes [3-5].

Despite the importance of CPD, its uptake is impeded by personal, professional, and environmental factors [6]. Previously reported barriers to participating in CPD have included time, access, and cost [7]. Clinicians have expressed reluctance to make time for CPD (due to the demands of busy caseloads, social life and family, and travel to education-delivery venues) and have noted constraints associated with the direct costs of CPD and indirect costs associated with lost earnings. These factors are amplified in regional and remote areas where workforce shortages can increase caseloads, and geographic distance renders CPD less accessible and more costly [8]. These constraining factors may have a negative effect on workforce satisfaction and make it difficult to retain staff in regional and remote areas. Clinicians in regional and remote areas of Australia have reported dissatisfaction with lack of professional development opportunities and the associated professional isolation, and these factors have been linked to staff recruitment and retention difficulties [9].

The traditional approach to CPD has been face-to-face education, often involving large live audiences (eg, seminars or conference presentations). This face-to-face approach may be difficult or costly to access for people who live a long distance from the education venue. Further to this, participants need to be able to allocate the requisite time in their schedule to enable participation. This mode of educational delivery can create a significant burden on educational institutions through administrative load and financial risk if the threshold for participant numbers required for financial viability is not reached. These are key factors that limit the implementation of training programs for health professionals in regional and remote areas.

Web-based CPD provides an alternative method of education with the potential to overcome many reported obstacles. Web-based delivery of educational content provides flexibility of access and promotes a learner-centered approach to learning, enabling interaction with learning materials at a time that suits the consumer [10]. Using the Web to deliver CPD requires the consumer to have Internet access, information technology literacy, and occasional technical support, as well as the evolution of teaching and learning resources that are tailored to suit the medium [11,12].

It is possible that multimedia or Web-based instruction may be inappropriate for teaching or monitoring practical skills [11-14]. Practical-skills competence requires a degree of self-evaluation that is often inadequate in the absence of appropriate feedback [15]. However, innovative design of feedback using interactive and collaborative Web-based technology might overcome the purported limitations [16].

Previous investigations of Web-based education have contrasted this with providing no education [17-27], rather than applying a head-to-head approach for comparing Web-based with face-to-face education. One report of an investigation comparing behavior change from Web-based versus face-to-face education found that Web-based CPD can produce changes in behavior in physicians working in lipid management, when practice change is measured using the number of requests for lipid management tests [28]. Gains in knowledge comparable with or superior to those obtained via live education delivery were also seen. It is plausible that Web-based delivery can also produce learning outcomes equal to face-to-face education when applied to the delivery of a complex hands-on clinical skill.

In this study we compared a Web-based approach to providing a CPD course versus a traditional “live” education-delivery approach in the context of education for the complex clinical skill of exercise prescription for falls prevention. Falls are a significant threat to the safety, health, and independence of older adults, accounting for more than half of the accidental deaths among older adults [29,30]. In Australia, the total cost of fall-related injury is expected to triple to A$1375 million per year by 2051 unless effective prevention or lower treatment costs occur [31]. Exercise is an effective intervention for the prevention of falls; however, some forms of exercises have been shown to be more effective than others, with systematic reviews and meta-analyses finding high-dose exercise, including challenging balance training, the most effective [32,33]. Several Web-based falls-prevention programs have been introduced [34,35]. Some exercise programs, such as the Otago program, have been the focus of Australian nationwide public health strategies to address the issue. Programs to date have focused on the provision of one single program, and not on teaching the learner the broad range of exercise prescription skills that would allow exercises to be designed, tailored, and applied by the clinician to those at risk of falls. Exercise prescription is a skill set that requires knowledge of anatomy, biomechanics, psychology, and the practical skills to safely guide the patient to achieve targets of improved balance, stability, risk reduction, and confidence with ambulation. Teaching practical skills such as guiding a patient to master exercise for the prevention of falls provides an important and clinically relevant context for investigating the relative effectiveness of Web-based and face-to-face modalities.

## Methods

### Design

This study was a randomized trial with concealed allocation and blind outcome assessment comparing two educational interventions (Web-based compared with face-to-face education in falls-prevention intervention) that employed a mixed (qualitative and quantitative) evaluation framework. Ethics approval was obtained through both the Monash University Human Research Ethics Committee and the Southern Health Ethics Committee.

### Participants

Participants were required to hold at least a bachelor’s degree in any health science and reside in the state of Victoria, Australia. Participants were invited through a recruitment information package consisting of an electronic flyer, explanatory statement, consent form, and registration form. The recruitment package was distributed by email through managers of target professional disciplines via the Victorian department of health email distribution channels and by direct contact with private practices and community health centers in the geographic areas, two regional and one metropolitan, where face-to-face delivery was scheduled. Registrations were accepted for a period of 6 weeks from the time of the mailout, closing 1 week before the intervention started (May 2010). Applications for registration were screened for evidence that inclusion criteria were met (bachelor’s degree in a health science and residing in Victoria, Australia).

### Interventions

The content of the interventions was informed by three scoping activities: a review of the falls-prevention program literature to establish common elements in existing falls-education programs; phone interviews conducted with 24 clinicians who were leaders in falls prevention to establish current practice in specialized clinics; and phone interviews conducted with six target audience representatives from multiple professions to identify the knowledge gap between expert clinicians and the education target audience. The course content then underwent an external review by a falls-prevention researcher, a clinical leader, an academic leader, and a member of the falls-risk target audience. All learning objectives for both interventions were mapped and linked to relevant resources and tasks, and were matched in content and time requirements. The learning objectives included the physiological principles of exercise prescription for falls prevention, assessment procedures, exercise selection and delivery, and techniques to encourage program adherence and behavior change. To enhance consistency of delivery and adherence to the planned curriculum, the face-to-face leader, his or her delivery assistant, and the Web-based facilitator were all trained from a single DVD, comprising the same footage and learning resources used in the construction of the Web-based program.

One group received face-to-face education by attending a 1-day (7-hour) seminar, facilitated by a local expert in this field who had practiced clinically in the area, and had published and completed a PhD in the arena of falls prevention. To assist student revision, a support package was posted to participants before the seminar started that included a copy of the presentation slides, reference to further readings, and a DVD of the assessment procedures to be covered in the seminar. The seminar was held outside usual work hours with participants allocated to the nearest program location.

**Figure 1:**
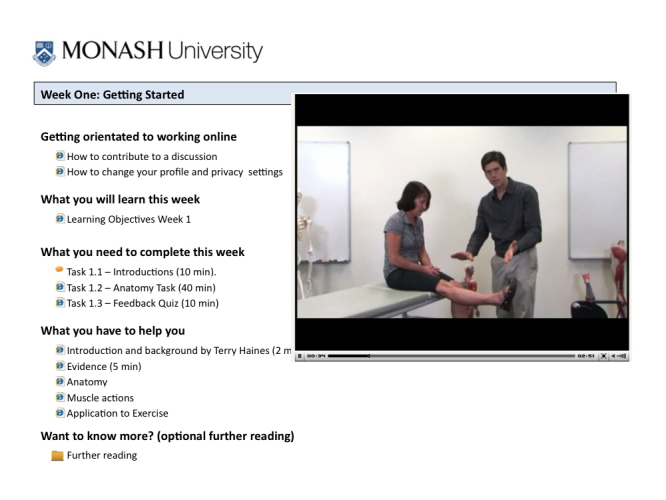
Screenshot of the constructed short-course home page, illustrating the typical integration of learning resources, activities, and supports. The segment of image on the right shows an example of a multimedia resource—in this case, a discussion on measuring quadriceps strength.

The other group took part in Web-based delivery of the equivalent educational material over a 4-week period (anticipated to require 7 hours total time commitment over 4 weeks) facilitated by a Web-based tutor who corresponded with participants through Web-based discussions and was available by phone if problems occurred. The Web-based course was constructed within the online learning system Moodle (Moodle.com, Perth, Australia), which uses open source code and is available in the public domain. Figure 1 shows a screenshot of the constructed course home page to illustrate the typical integration of activities and learning resources. Participants were posted a DVD comprising the multimedia used in the Web-based program as a troubleshooting solution if they encountered difficulties viewing the content online. Participants were allowed to progress through the program at their own pace, completing educational activities any time during the 4 weeks. Learning tasks ranged from self-directed reading and formative quizzes to interactive skills-practice sessions with feedback opportunities. For feedback, students uploaded digital footage of their skill mastery, which was viewed by the Web-based tutor. They were then guided through a reflective task by reading the tutor’s comments of typical group performance in the task submissions, and they could view a tutor-selected exemplar of student performance to enable benchmarking of expectations of performance competency.

### Outcomes

Kirkpatrick’s hierarchy of educational outcomes proposes that training effects be examined for four levels of impact: (1) participant reaction, (2) participant knowledge, (3) participant change in behavior, and (4) change in health outcomes [36]. Outcomes were measured across levels of impact 1 to 3, with the same measures of both groups taken at the same time. Level 1 outcomes were measured through program attendance and adherence (assessed by signatures on a register of attendance for face-to-face delivery and computer-generated usage reports for Web-based participants), self-reported satisfaction (assessed via an electronic survey emailed to both groups), participants’ ratings of the relevance of the program content to their current work roles, and self-reported estimates of time spent engaged with the learning resources. Level 2, knowledge, was measured via a 1-hour knowledge test, conducted approximately 1 week after completion of the program, and an assignment submission requiring a description of an exercise program tailored to a hypothetical client scenario. Level 3 outcomes were measured by self-reported change in practice, including a self-report of whether participants had changed their practices since completing the program. Change in practice was first measured through asking participants “Since completing the program, have you changed any aspects of how you manage your falls and balance clients?” and then, by open text comment, “If you answered ‘yes’ to the question above, please indicate in the space provided below in what way the program has changed your management of falls and balance clients.” This question was then followed by a series of closed questions asking participants to provide their opinion about specific aspects of exercise prescription, such as “I attempt to use more motivational interviewing techniques.” Participants responded to these closed questions using a Likert scale with five response categories (strongly agree, through to strongly disagree). Participants could not see these specific items until after they had completed the open-ended question so that these items would not influence the response to the open-ended question.

### Procedure

Randomization was stratified by professional group (physiotherapist, occupational therapist, nurse, exercise physiologist) and nearest live program-delivery venue. An independent research assistant recruited participants in order of receipt of their registration and was unaware of group allocation. For the random allocation of participants, a computerized random number sequence was generated using permuted blocks of two, four, and six participants and stratified by geographical location of recruitment. Examination scores were automatically corrected using the online learning system that delivered the examination to both groups. Assignment submissions were de-identified before being forwarded to a blinded assessor. Assignment submissions from both groups were scanned and reprinted to remove potential visual differences. The independent assessor, blind to the group allocation, assessed the assignments against pre-prepared assessment criteria. Thematic analysis of open text responses was verified by two researchers, with any lack of consensus referred to a third reviewer.

### Statistical Analysis

Demographic characteristics of the two groups were compared at baseline using the chi-square test for items with binary data, and a 2-sample *t* test for comparing items with continuous data. Survey questions for measuring Kirkpatrick’s hierarchy domains *participation reaction* and *change in behavior* were custom developed by the investigators based on a previously published description of these domains [36]. Responses to these items were subjected to principal-factor factor analysis with rotation. Four factors identified with eigenvalues greater than 1.0 were then reviewed for consistency and redundancy. Items were removed from factors if their rotated factor loading was less than 0.5 or the item uniqueness was less than 0.2. We then reviewed the factors generated for coherence and plausibility, which led to the removal of one factor, as the two items that it was composed of were seemingly unrelated. The remaining three factors were named based on the included items. These factors were (1) satisfaction with content and its relevance, (2) satisfaction with the support and facilitation, and (3) change in clinical behavior. Cronbach alpha for each factor was .92, .77, and .84, respectively. These factors were considered to be consistent with the theoretical framework used to originally guide item development. A simple summative score was then constructed for each factor following the principles of classical test theory. The resulting summative score represented the domain named by that factor. The wording of items contained in each factor is presented in Multimedia Appendix 1. The scale score range for each factor was 0–35, 0–15, and 0–30 respectively. The effect of education-delivery approach on each factor was examined by linear regression using the factor score as the dependent variable and a dummy variable coding for education approach (0 = face-to-face, 1 = Web-based) as the sole independent variable in the model.

Knowledge test and assignment scores were marked out of a score range of 0 (no correct answers) to 30 for the knowledge test and 100 for the assignment, before being converted to a percentage. These scores were examined for normality of distribution by examination of histograms and tests for skewness (skewness = –0.56, *P* = .02, indicating that the null hypothesis that the data is normally distributed can be rejected [37]), where a high proportion of scores were loaded toward the maximum score. We therefore used the nonparametric rank sum test (Wilcoxon test or Mann-Whitney *U* test) to compare groups on this outcome. The amount of time individuals spent engaged with optional further reading materials was also found to be skewed (skewness = 4.00, *P* < .001); hence, we also used a rank sum test for comparison between groups.

Responses to open-ended questions were analyzed thematically by two investigators. We used the statistics software package Stata version 11 (StatCorp LP, College Station, TX, USA) for all data analyses.

## Results

**Figure 2:**
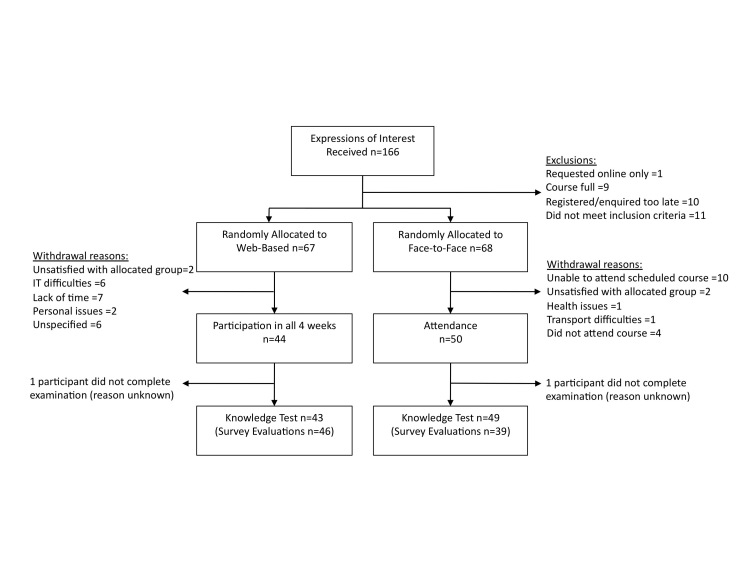
CONSORT flowchart showing attrition of study participants (IT = information technology).

Of the 166 participants initially screened, representing a range of professions including physiotherapy, occupational therapy, exercise physiology, and nursing, there was gradual attrition from randomization to participation in the trial (n = 67 Web-based, n = 68 face-to-face), to completion of the educational content (n = 44 Web-based, n = 50 face-to-face), to completion of the posteducation knowledge test (n = 43 Web-based, or 36% attrition from initial random allocation, and n = 49 face-to-face, or 28% attrition from random allocation; Figure 2).

Participant demographics are presented in Table 1 and indicate a relatively even distribution of baseline characteristics.

**Table 1:** Demographic characteristics of study participants by group

Demographic item		Education mode		*P* value
		Web-based	Face-to-face	
Gender (male), n (%)^a^		10 (22%)	7 (18%)	.43
Previous falls research participation, n (%)^a^		2 (4%)	5 (13%)	.18
Previous falls publication, n (%)^a^		1 (2%)	0 (0%)	.35
Previous falls professional development, n (%)^a^		11 (23%)	10 (25%)	.85
Profession, n (%)^a^				
	Occupational therapy	5 (11%)	3 (8%)	.97
	Physiotherapy	26 (57%)	20 (51%)	.93
	Nursing	10 (22%)	11 (28%)	.95
	Exercise physiology	4 (9%)	4 (9%)	.96
Years since qualification, mean (SD)^b^		4.17 (1.75)	4.15 (1.56)	.66

^a^ Chi-square test.

^b^ Two-sample *t* test.

### Kirkpatrick’s Level 1: Reaction

Mean (SD) scores in the domain *satisfaction with content and its relevance* appeared to be similar for the two groups: Web-based, 25.73 (5.14), and face-to-face, 26.11 (5.41). Comparison between groups using linear regression for this domain did not identify a statistically significant difference: beta coefficient (95% confidence interval [CI]), –.38 (–2.70 to 1.94); *P* = .75. Mean (SD) scores in the *satisfaction with course facilitation and support* were also comparable between groups: Web-based, 11.61 (2.00), and face-to-face, 12.08 (1.54). The difference was also not statistically significant: beta coefficient (95% CI), –.47 (–1.29 to 0.35); *P* = .25.

Web-based participants reported spending a mean (SD) of 8.7 (6.6) hours engaged with the compulsory learning resources as compared with the 7 hours required of the face-to-face group, which was controlled by the venue’s opening hours. Web-based participants reported spending significantly more time engaged with the additional learning materials than the face-to-face group: median (interquartile range [IQR]), 1.0 (0.8–2.0) hours compared with 0.0 (0.0–1.0) hours; rank sum *P* = .002.

### Kirkpatrick’s Level 2: Knowledge Outcome

Knowledge test results were comparable between face-to-face and Web-based groups: median (IQR) for Web-based, 90.00 (70.89–90.67), and for face-to-face, 80.56 (70.67–90.00); rank sum *P* = .07. The median (IQR) scores for the exercise assignment were also comparable: Web-based, 78.6 (68.5–85.1), and face-to-face, 78.6 (70.8–86.9); rank sum *P* = .61.

### Kirkpatrick’s Level 3: Change in Practice

Mean (SD) scores in the domain *change in clinical behavior* were similar between groups: Web-based, 21.75 (4.40), and face-to-face, 21.88 (3.24). Comparison between groups using linear regression for this domain did not identify a statistically significant difference: beta coefficient (95% CI), –.13 (–1.99 to 1.74), *P* = .89.

Thematic analysis of the optional open text comments by participants revealed that Web-based participants were primarily disclosing changes in their application of motivational interviewing strategies (8/22), along with changes to improve the competency of their client assessments (8/22). In contrast, participants in the face-to-face mode of delivery did not comment regarding motivational interviewing (0/21), with change in assessment (10/21) and change in exercise prescription (12/21) as the two components of practice most frequently identified.

Of the Web-based participants, 24% (11/46) reported being apprehensive about undertaking a Web-based program before the program commenced, rating agree or strongly agree to the statement on a 5-point Likert scale ranging from strongly disagree to strongly agree. After the program had been completed, 70% (32/46) of respondents indicated that they quickly became accustomed to the Web-based environment and 80% (37/46) indicated agreement that they would be willing to undertake another Web-based program.

## Discussion

### Principal Results

Web-based and face-to-face approaches for providing training in the field of exercise prescription for falls prevention produced comparable results for all three levels of Kirkpatrick’s hierarchy of educational outcomes. Previous studies have reported that Internet-based CPD can increase the acquisition of health professional knowledge and lead to change in clinical behavior in single-profession educational studies [28]. Previous head-to-head studies of Web-based versus live delivery of CPD have examined procedural and theoretical skills applied to decision making, such as recognizing the need for referral for further medical testing. This study provides evidence that outcomes of Web-based CPD are not significantly different from those of face-to-face CPD applied to an interprofessional audience and a subject matter that encompasses a broad range of practical skills including clinical decision making, hands-on skills, and high-level communication.

With comparable results between delivery methods, practical considerations should arguably drive the choice of delivery mode. These may include being able to create a more standardized educational product that is less influenced by the style, experience, and knowledge of individual presenters, and the ability to protect corporate knowledge with changes in personnel, or the educational institution’s information and communication technology capabilities, resources, and support structure.

This study has demonstrated that, in the field of falls prevention, Web-based education may provide results in terms of Kirkpatrick’s levels 1 to 3 equivalent to those of face-to-face education. This enhances the capacity for upgrading the skills of health professionals for whom geographic isolation is a barrier to participation in CPD in this area. With greater skills they may be able to better meet the needs of older adults at risk of falls residing in rural and remote areas.

### Limitations and Future Directions

Some strengths and limitations may have affected the collection, analysis, and generalizability of the intervention results.

Limitations in data collection may have arisen from the custom design of the survey questions intended to measure the Kirkpatrick hierarchy domains. The examination was open book, although no overall grade was awarded to the student for the short course, so as to minimize the motivation for collusion and looking up answers from alternative sources. We were unable to control for collusion between participants in either the Web-based or face-to-face intervention group, as the examination was conducted online and off-site.

We noted moderate correlation between the knowledge test and assignment scores (Pearson *r* = .44); however, this is not surprising considering that the intent of the exam was to test memory of theoretical knowledge, and the assignment was to test applied clinic-based problem solving and ability in exercise design.

Supporting positive changes in Kirkpatrick’s hierarchy level 1 may have included program design aspects aimed at decreasing the transactional distance, or feeling of isolation and separation from the learning group, through an actively engaged facilitator, available discussion forums, and multiple tasks encouraging participant networking. We provided numerous opportunities for feedback on knowledge and practical skills, through weekly knowledge tests with automated feedback, video-skill submissions with feedback, and selected peer submissions published through the online learning system, to allow active reflection and self-evaluation of skills mastery. Participants were additionally supported by an extensive information technology support network provided by the administering university, with helplines available from 8:00 AM to 8:00 PM Monday to Friday. Factors that may have negatively affected satisfaction and attrition may have included nursing participants indicating that the program skills and objectives were not as relevant to their profession as to participants with physiotherapy qualifications. Information technology difficulties were also experienced as a direct result of complications arising from the process of randomly assigning participants to Web-based or face-to-face delivery and through upgrading of the program administrators’ skills in the first iteration of the program. It is anticipated that many of these difficulties could be eliminated in future modeling of the program. The program was offered to participants at no cost, which decreases the participants’ commitment to the program and negatively affects attrition [38]. The program design required a significantly high level of information technology literacy by the participants. Although clinicians’ use of the Internet and other information technologies is increasing exponentially, with between 78% and 85% of clinicians accessing health information via the World Wide Web [39], universal access and competency in information and communication technologies cannot be assumed [11]. Contemporary university students, of generation Y, are generally computer literate and embrace new technology [40]. However, given that the participants in this trial were postgraduates, of generation X, with a mean of 3.9 years since obtaining their professional qualifications, we did not know whether participants would be able to readily source and manage information technology requirements, particularly with tasks involving electronic submission of self-videos. Although 70% of respondents reported that they quickly became accustomed to the Web-based environment, only 80% of Web-based participant respondents reported that they would complete another Web-based program.

The follow-up regarding clinical practice change was limited to a short-term follow-up. It is feasible that participants ceased using these skills in the medium or longer term, or conversely increased their use of these skills. It is unknown whether patients of participants had health outcomes that were improved to a greater or lesser extent as a result of the participants’ educational approach. We excluded, but could have included, some health professional groups from the present study that could have a role in the clinical management of older people at risk of falls.

This study also has limitations in how broadly these results can be generalized into other CPD areas. The content focused on one clinical area, and it is possible that other subject areas may not be as readily adapted into a Web-based format (for example, teaching spinal manipulation techniques).

Extensions of this research could include investigations of cost minimization, factors influencing willingness to enroll in Web-based education, and economic efficiency of face-to-face and Web-based approaches to CPD. If Web-based education proved to be a more efficient method for increasing the number of skilled clinicians in this area, yet comparable in its educational outcomes as shown in this study, then this may influence public health strategy in addressing falls in our community, and have wider implications still on how CPD is delivered around other health priorities. Another extension of this research would be to assess the impact of program participation on health outcomes, addressing level 4 of Kirkpatrick’s hierarchy.

### Conclusions

Face-to-face and Web-based approaches to the delivery of education to clinicians on the subject of exercise prescription for falls prevention produced equivalent results in all of the outcome domains, with the exception of Web-based participants reporting more time engaged with the optional learning resources. Practical considerations should arguably drive the choice of delivery method, which may favor Web-based provision for its ability to overcome access issues for health professionals in regional and remote settings.

## Multimedia Appendix 1

The wording of items contained in each factor.

## Abbreviations

CPD: continuing professional development
IQR: interquartile range
